# A time for new north–south relationships in global health

**DOI:** 10.2147/IJGM.S146475

**Published:** 2017-11-07

**Authors:** Jin Un Kim, Obinna Oleribe, Ramou Njie, Simon D Taylor-Robinson

**Affiliations:** 1 Division of Digestive Health, Department of Surgery and Cancer, Imperial College London, London, UK; 2 Excellence and Friends Management Care Centre, Abuja, Nigeria; 3 MRC, Serekunda, The Gambia

**Keywords:** global health, bioethics, clinical trials, Africa, exploitation, imperialism

## Abstract

The modern concept of globalization in health care and clinical research often carries a positive message for the “Global South” nations of Africa, South America and Southeast Asia. However, bioethical abuse of participants in clinical trials still exists in the Global South. Unethical studies directed by the “Global North”, formed by the medically advanced nations in North America, Western Europe and Japan, have been hugely concerning. The issue between the Global North and South is a well-recognized socioeconomic phenomenon of globalization. Medical exploitation has its roots in the socioeconomic interactions of a postcolonial world, and solutions to reducing exploitation require a deeper understanding of these societal models of globalization. We explore the fundamental causes of imbalance and suggest solutions. Reflecting on the globalization model, there must be an effort to empower the Global South nations to direct and govern their own health care systems efficiently on the basis of equality.

## Introduction

Ethical negligence in human research has received global attention since the Nuremberg trials of 1947, which with other unethical trials highlighted the importance of human bioethics to a worldwide audience. International guidelines, such as the Declaration of Helsinki, have been implemented as a response, but the issue remains topical within the practices of developing regions, or the “Global South” – a term that we will use, referring to the many resource-limited nations in Africa, Southeast Asia, South America and parts of Europe.

Global health has been central in assembling health incentives from an international perspective, as the “study, research and practice that places a priority on improving health and achieving equity in health for all people worldwide.”^1^ In addition to resource mobilization, there has been remarkable expansion of clinical trials performed in developing countries.^2^ This global movement of research is very much comparable to economic globalization models of cost-benefit with the Global South nations offering lower salaries for human labor and shortened timelines for clinical testing.^3^,^4^ Moreover, a fundamental incentive for the geographical shift in research derives from more tolerant regulatory and ethical barriers than in Western Europe, North America and Japan. This ethical laxity has created exploitation of human subjects and led to events that bear, at least in part, some uncomfortable resemblances to those practices that saw the light of day at the universally condemned Nuremberg trials.

### Purpose

The problem of human bioethics in developing countries is multifaceted, many of its causes being deeply rooted in their historical–geopolitical interactions. First, we aim to explore the reasons predisposing to bioethical dilemmas by defining basic globalization concepts and colonial theories. Next, we wish to highlight the areas that cause, or are directly contributory to, unethical practices in medical health care and clinical trials in the Global South. Finally, we wish to propose solutions to each identified issue to, ultimately, reinforce partnership between the Global North and South on the basis of mutual interest and equality.

## The new era of imperialism

The first scholarly use of “globalization” was in 1960 and was used to portray the growing trend of intercultural interactions.^5^ Although this was a novel concept at the time, it depicted a phenomenon of economic, sociopolitical, informational and ideological integration that had long existed under the contextualized term “imperialism.” The optimistic values that globalization endeavored to represent were implemented, not so long ago, as “an unequal human and territorial relationship, usually in the form of an empire, based on ideas of superiority and practices of dominance, and involving the extension of authority and control of one state of people over another.”^6^

The incentive behind imperialism first is of perceived or want of superiority, and as a direct result, that of justified dominion. Schmitt^7^ describes the imperialistic movement much in parallel with the prerogative of a king, where the state or power establishes itself above the law, as the exception. Specific examples can be derived from the British Empire’s ideologies, which can be traced back to the concept of translatio imperii (transfer of rule) that contended the translation of global political power from Greece to Rome, and then to Western Europe, being called upon as a justification of European imperialism.^8^ Again, studies such as Cannadine’s^9^ *Ornamentalism* reported that there was no racism in the British Empire, simply that the British imperialistic perspective was a medium of liberation, as the rightful mother nation. Attesting to today’s definition of globalization, imperialism had been implemented and practiced in an increasingly global field from the archaic Greek and Roman Empires, through the Mongolian and the Ottoman Empires in the 13th century to the British and the French Empires of the 19th and 20th centuries, and latterly to the Soviet Empire. Only after the Second World War, imperialism, in the traditional sense, had ended to inaugurate a new age of international interaction.

### Tropical medicine in the colonial world

There is no doubt that even the medical discipline evolved as a direct consequence of Western imperialism. This evolution, namely the advent of tropical medicine, was perhaps inevitable due to the sudden integration of colonial settlers to a new land of “tropical diseases.” It is interesting to note that while tropical medicine has become an essential aspect of modern medicine, the attitude on which it was created was part and parcel of the colonial agenda. Historical narratives, which focus on tropical medicine and its advancement during the colonial eras, often hold it as a tale of “triumph of disease; the picture they portray is of ‘diseased natives’ made well by white man’s medicine.”^10^,^11^ Moreover, according to Worboys,^12^

the investigation and teaching of the etiology and treatment of tropical diseases was developing in an environment and culture totally different from the tropics. Work on etiology became exclusively scientific, based upon parasitological studies and the germ therapy of disease. The clinical treatment of these diseases took precedence over prevention and epidemiological studies on disease incidence and control.

Be it for the treatment of disease or for scientific knowledge, tropical medicine was first and foremost an asset of the colonial rulers.

## Medical neocolonialism

The natural expectation for a previously colonized territory, once the colonizing power is abolished, is the reinstatement of its international sovereignty, as it was before the events of colonization. Rarely, however, does the state receive its rightful postcolonial integrity, and instead, the influence of the former colonial powers is carried forward, so much so that the liberated nations remain very much dependent on their mother state. This is a phenomenon described by the late president of Ghana, Kwame Nkrumah, as “neocolonialism.”^13^ The imbalance of benefit and compromise once seen in the colony is thus unchanged in the theory of neocolonialism within the framework of future postcolonial interactions. This particular idea of sustained exploitation has been coined the “dependency theory.” The theory lends itself to the idea that extraction of human and natural resources of peripheral, or poor, countries flows directly into the wealthy countries at the center, resulting in “foreign capital [being] used for the exploitation rather than for the development of the less developed parts of the world.”^13^ If this idea is extrapolated to the current global picture of inequality, it suggests that the problem of global poverty is artificial and is perpetuated by international relationships and gives support to the original Marxist analysis that “the underdevelopment of the Global South is a direct result of the development in the Global North.”^14^

In addition, beyond and above the temporal concept of a period after events of decolonization, a relatively novel concept of “post-colonialism” describes the sociopolitical, economic, cultural and intellectual hybridity of the people residing in decolonized states. The postcolonial theory accounts for the development of a new identity of the colonized person, as determined by the racism and subjugation that fundamentally arise from the construct of a colonial society. Furthermore, the postcolonial state extends to the mother country, and even after the events of decolonization creates homogenizing, binary boundaries for areas once colonized. This has given rise to over-inclusive and arbitrary descriptive terms in Global North cultures, such as the “Third World,” and the “Orient.” In essence, the previous role-play of the colonial power and the colonized nations continues far beyond formal colonialism, through sustained international influence and attitude.^15^

The residual socio-politico-cultural effects of colonialism are evident in the postcolonial nations. Moreover, the events of globalization are perpetuating these tensions, an Indian research unit observing that “the great range of actual measures carried on under the label of globalization[…] were not those of integration and development. Rather, they were the processes of imposition, disintegration, under-development and appropriation,”^16^ resulting in economic and political dependence, and continued rape of national resources by the Global North. The neocolonial and postcolonial framework of globalization discussed earlier unfortunately also manifest in global health practices.

Multiple interplaying factors remain responsible for the shortcomings in international human research, and each constitutes a complex set of issues that must be addressed holistically.

### Ethical oversight: encouraging ownership of ethical standards

Oversight of the conduct of human trials safeguards the rights, health, economic well-being and welfare of participants throughout the entire research process. Clinical trials that have been designed by the Global North, for the Global South, however, have too often shown substandard oversight in comparison to the degree of fidelity implemented in their own countries, for their own subjects. Perhaps the case that sparked the most controversy, and in turn was the most revealing of the practices of medical neocolonialism, involved the “no-treatment control” group in the zidovudine trials in HIV-infected women in Asia and sub-Saharan Africa, despite the availability of zidovudine and its proven efficacy for reducing vertical transmission of HIV from 25.5% to 8.3%, published by the AIDS Clinical Trials Group (ACTG) in France and the USA.^17^ After the initial success of the ACTG study, the World Health Organization (WHO) made decisions to drastically reduce the cost of future trials using zidovudine, by implementing a much simpler placebo-controlled design in 15 of 18 trial populations in the Global South. Once the information became public in 1997, unsurprisingly, the topic of human rights violation in developing countries became headline news in the scientific communities.

The prolonged debate that followed addresses interesting points. First, it raises the issue of ethical relativism in “standard (medical) practice.” The fundamental justification made by the panel of experts responsible for the placebo-controlled design was the concept of adhering to the “local standard practice,” which in the case of poverty-stricken regions was undoubtedly a “no prophylaxis medical care.” Many authors, however, responded with heavy criticisms. The response mentioned that the placebo-controlled study design was suboptimal under the circumstances, as equivalence or non-inferiority designs may have been implemented instead. Lurie and Wolfe,^17^ who spearheaded the attack, also stated that the phrase “standard practice,” which was preached to justify the study, was a misunderstood concept. The lack of HIV prophylaxis in resource-limited regions was, and still is, a fundamental issue of inaccessibility, not a consideration of alternative treatments or of previous clinical data, simply meaning that these situations are not local “standard practices,” rather they are a manifestation of the Global North’s willingness to lower the clinical standard in the Global South. Turning the statement around, they revealed the powerlessness of the countries of the Global South, and the subsequent domination by the Global North, much in parallel with other forms of neocolonial exploitation. What informed these decisions – economic or scientific reasons?

Second, this study raises the issue of poorly structured review boards in developing countries that are either inadequately trained, non-independent or altogether nonexistent. A recent review of ethical committees in 33 African regions revealed that more than half of these were formed after the year 2000. The reviews also revealed that only 34% of the committees included individuals who had knowledge or training in medical ethics, and not all the committees provided continued training or adequate remuneration for those board members.^18^ With little training and lack of financial recompense for the hours worked, the quality of many of these committees may at best be considered contentious, which again emphasizes the potential for exploitation by a global pharma incentive.

Finally, this debate represents the fundamental issue of scientific exploitation within developing countries. Looking at the clinical trial in question again, zidovudine had been previously successfully tested in the USA and France. The drug was found to reduce the incidence of HIV infection of the newborn by two-thirds at the first interim analysis, at which point the study was terminated and zidovudine was swiftly implemented as a recommended regimen for all HIV-positive pregnant women.^19^ Owing to the expense of the regimen, subsequent study designs were conceptualized to determine whether the regimen dosage could be lowered, while still providing similar outcome. The placebo-controlled design of the follow-up trials pays yet further homage to the theory of neocolonialism in global health medicine. These follow-up studies were implemented in the Global South, the placebo group being neglected of available treatment – an outrageous notion given the interim termination of the original US-based study.

Solutions that address such neocolonial exploitation require, in essence, an ethical oversight system that is on par with that of the Global North. Most importantly, the system must be stringent and independent, in the sense that it makes sound judgment according to the global guidelines, while remaining impartial to external influences. Tackling globalization, or the effect of it, requires each individual nation to take the reins of sound leadership. Of course, it may initially require help from outside nations, or the lead taken from the WHO or the World Health Assembly. The relationship should not represent that of servitude.

An institutional review board (IRB) or research ethics committee (REC) is an independent committee designated to approve and monitor human research according to international bioethical guidelines. These often consist of a pan-disciplinary panel, covering multiple scientific, ethical and social professionals who conduct risk–benefit analyses to identify and exclude study designs that may expose their subjects to harm. However, it has been observed that the system of independent review centered on “impartiality” is frequently wrought with subjectivity and influence from stakeholders and researchers.^20^ The stakeholders may include government bodies, funding and academic institutions, each with their agendas. The regional ethical committees in the Global South may experience pressure from these stakeholders, which again, paints a picture of neocolonial devotion of the Global South to maintain a positive relationship with the Global North, even at its own expense. Furthermore, to answer the basic questions surrounding IRB efficacy, appropriate outcome measures must be defined. Fundamentally, this is a challenge in itself, as human welfare is a broad concept with multiple measurable indicators.

What is required, then, is both a local and a global approach to applying safeguarding measures through ethics committees. Similar to the review of IRBs mentioned earlier, in a separate case study of the structure of 12 ethics committees in Africa, it was found that there were fundamental issues with regular meetings, adequate and repeated training, procedural and administrative handling and finances.^21^ The key issue of funding and training is difficult to overcome, even with international aid and sponsorship. Therefore, we propose the long-term development of an international review board for randomized clinical trials (RCTs), perhaps under the auspices of the WHO, where there are representatives from a continental ethical body, such as those from Africa or Southeast Asia, who may attend to deter unethical trials from being approved. This may facilitate legitimate collaboration between different countries, and representatives from the Global South may receive the same training and privileges. The stringent approach is likely to improve both the quality of human subject research performed in developing countries and the scientific interrelations between the Global North and South.

Before global collaboration can take place, it is necessary to have a targeted approach in establishing an IRB where there is none available or optimizing an existing group to acceptable standards. A systematic review of this study has identified several key areas that may measure IRB efficacy from a welfare perspective.^22^ These include health-related outcomes of research participation and protection of human rights. In addition to these primary outcomes, the IRBs should be assessed on the logistical aspects, such as the group composition, types of studies reviewed, review times and the decision-making process. Admittedly, these changes will be difficult to implement for the countries themselves, given the lack of health infrastructure in the developing Global South. However, with the correct aid given by the Global North, these changes are feasible, and it is encouraging to note positive evidence of global funding in bioethical training in Africa, with a recent report showing that, post training, respondents were more likely to serve in international committees and on IRBs.^23^ In the interest of the global research community and the bioethics of human participants, it may be helpful for the international committees to support the self-determining aspect of the Global South before advancing with its own research agendas.

In addition, on a more individual level, a clear understanding of the physician’s own duty may be required and encouraged in the Global South. A recent study in the United Arab Emirates stimulated discussions based on the definition of professionalism, not as dictated by western vantage and philosophy.^24^ In view of the idea that professionalism is connected to a society’s culture and values, an adoption of the fundamental cornerstones of local societal models, such as religion and cultural identity is necessary to enhance self-determination of health care workers in the Global South. By enhancing the link between the expectation of professionalism and of their intrinsic values, cultural autonomy may be the deterrent that stands against conformation to international frameworks, which may demand exploitation of their people.

### Contextualizing consent

Informed consent is central to human research. A thorough consenting procedure acts as a barrier to harmful bioethics and empowers participants with choice of and within the trial. The neocolonial world undermines informed consent in two main ways – by misguided information or by limiting participation freedom. Before delving into the importance of contextualized consent, there must be an appreciation of the difficulty of applying informed consent, as it is universally understood, in a globalized medical setting.

First, numerous groups in the Global South will identify with a “communitarian” approach to human rights, which is applicable in the setting of a research trial. That is to say that their communal societal structure means a “person’s dignity and honor flow from their transcendental role as a cultural and social being,” thus “rights” are understood in a collectivist, rather than an individualist sense.^25^ The decisions are often overseen by the head or the elder of the village or tribe. Even when the members are agreeable to the decision made by the head of the community, this negates the value of independent, autonomous decision making that is required in informed consent.^26^ Some critics argue that despite this issue, it is important to observe their customs and reduce the Global North bioethical transposition, or equally, to respect ethical relativism in applying international guidelines to a particular cultural setting.

Hellsten^25^ specifically argues the point that, in African communities, the approach to human rights is communitarian, not Marxist utilitarian, where public interest may subsequently suppress individual rights. The relativistic argument demands judgment of intent and action in a contextualized setting, as well as tolerance toward widely varying cultures, especially if they present with logical justifications, as that of Hellsten.^25^ This is a noble sentiment, but from a practical perspective, ethical relativism, and even accepting the African interpretation of the concept of collective human rights, is highly problematic. When there is no overriding set of moral guidelines, there is little support to resolve conflicts during the inception of study designs, and the ethical justifications are ultimately based on individual value judgments that are overwhelmingly subjective.

Cross-cultural ethical standards, therefore, must revolve around an absolutist system, but one that includes respect and sensitivity to the cultural difference of the Global South nations, whatever the constitution of respect may be in the particular setting. Moreover, this gives scope for corruption in the name of relativism. If the fate of a group is in the hands of a corrupt few – that is, in this scenario, the heads of communities – then respecting cultural relativity may lead to unwanted harm of the unsuspecting members of the community. When this simple picture of a village head is amplified to that of a corrupt national governing body, no doubt with the persuasive help of multinational companies and powerful corporations, the potential for harm becomes stark. This, therefore, highlights the importance of maintaining absolute ethics, thereby giving power to the individuals rather than paying homage to cultural diversity in these instances.

In addition, there must be a shift in the primitive Global North perspective, of homogenizing developing world cultures. There are thousands of different ethnic groups within Global South nations, all of which are undergoing dynamic changes through urbanization and industrialization. An argument can be made that the Global North approach to bioethics and first-person consent is becoming more relevant in the Global South nations, and greater integration of social expectation must be encouraged, rather than dwelling on the idea of ethical relativism.^27^ Furthermore, one must be mindful of the great variety of subcultures that exist even within Global North nations. The barriers of miscommunication and differences in the understanding of health and disease are evident in these contexts. Yet, the peoples of the coexisting subcultures in the Global North are not subject to ethical relativism, as this is a presentation of a bigger societal problem related to health literacy, rather than inherent in ethnicity and different cultural backgrounds.

The absence of medical options readily creates confusion between what is “research” and what is “treatment” among trial participants. Although this is an issue not limited to the Global South, it is clearly revealed by a series of interviews held during a HIV trial in the Ivory Coast (Côte d’Ivoire), where the reason for enrollment for one participant was the offer of “free health care and a hope to shield [them from disease].”^28^,^29^ The hope that being involved in a trial, and any trial at that, may then cure all the ailments of the participant, creates a pool of willing volunteers who, despite attempts at acquiring an “informed consent,” will enroll on the basis of misguided hope. It creates an extremely simple study population for the multinational drug companies to use. A fair argument was made by Annas and Grodin,^28^ who stated “researchers should presume that valid consent cannot be obtained from impoverished populations in the absence of a realistic plan to deliver the intervention to the population,” and argued that the role of informed consent in protecting participants is compromised when they are offered what appears to be medical care otherwise unavailable to them.

The importance of gaining an independent consent, thereby providing protection to individuals, is of utmost importance. Respecting cultural sensitivity is a desirable trait in researchers and ethics committees, but it alone cannot justify an unethical study design. To encourage scientific entitlement and hence, adherence, international health groups must work closely with the Global South nations to improve the overall health literacy of the population through means of effective and persistent education. Bioethical protocols and infrastructure have little value if they cannot be appreciated and understood. Therefore, a population-wide improvement in the awareness of health-related issues should be the prerequisite for the application of the international bioethical codes and practices. Once implemented, the issue of consent should be performed on an individual basis, but in a manner that respects the ethical values of the community, as not to dehumanize or undermine dignity or self-esteem.

However, altering cultural practices is never an easy task, even when it will ultimately result in the safeguarding of research participants in the Global South. Addressing the issue requires a more indirect and arguably holistic approach by improving health literacy and social capital on a population scale. Health literacy describes the skills that individuals utilize to effectively function within a health care setting, while social capital describes features of social organization. Unsurprisingly, systematic reviews show associations between low health literacy and poor use of health care systems, with poor or incorrect adherence to medical regimes, leading to adverse health outcomes.^30^ These skills reflect the importance of interpreting written and numerical information and engage in effective communication, as well as the more social aspects of collaborating in social organizations for mutual benefit. To address the issue fully in the developing Global South, the problem needs to be correctly identified with epidemiologic studies of communities. Although “health” and “literacy” are both dynamic terms, several measurable methods have been proposed, including the ease of access to health resources, the dominant health culture and how health knowledge is sought and shared between the peoples.^31^ Incorporating health-related education at an individual, community-based and governmental level is vital to empower individuals to make independent and informed decisions about their health and involvement in clinical research, ultimately closing the gap between the individuals’ autonomy and freedom.

### Appropriate research agendas and funding

The expansion of research in the developing world is often regarded as a promising solution to many of its local health concerns. However, the relevance of some clinical trial designs in the Global South has been uncertain. Glickman et al^2^ observed that many of the US-funded Phase III clinical trials in the Global South were for allergic rhinitis and overactive bladder, rather than imminent health issues such as malaria, tuberculosis and neglected tropical diseases. While it can be argued that these studies bring about global medical benefit, it is difficult to justify the true social value of conditions that are not imminent health concerns of the Global South. Moreover, drugs and pharmaceuticals to be used primarily in the Global South may never be tested in the Global North.

Even in the Global North, translating research outcome to clinical practice is a complex and imperfect procedure. This issue is accentuated in the Global South with poor infrastructure, lack of adequate human resources for health, and inappropriate funding. To ensure maximum benefit from clinical trials involving human subjects, adequate planning must occur from the conceptualization of design to the availability of the research intervention. The condition to be researched must be a fair and justified representation of a concerning medical issue that is relevant to the specific population involved in the trial. This invariably places greater importance on issues such as malnutrition, infectious diseases, genetic problems such as sickle cell diseases, noncommunicable diseases such as hypertension and diabetes and cultural malpractice, which have significant contributions to mortality and morbidity. Furthermore, an increased transparency in the publishing of epidemiologic and clinical data is required to characterize study populations, with the overall aim to select future study proposals that will have the greatest impact on a population. We need not look further than the outcome of the Millennium Development Goals (MDGs) to reinforce the importance of data transparency. Although this international effort provided the world’s only time-bound and quantifiable targets for addressing extreme poverty in the world, the outcome in 2015 revealed numerous limitations. The implementation of targets fundamentally requires true and validated baseline data. Many MDG countries, including those of relative wealth such as Nigeria, lacked any verifiable public health dataset, resulting in nonmeasurable outcomes for a widely anticipated global health project.^32^ Similarly, to encourage selection of appropriate research trials to take place in the Global South, there must be an effort to establish the population characteristics through large epidemiologic studies and consensuses.

Studies performed on well-characterized individuals may also be extremely useful if there is a degree of homogeneity between different population groups, allowing for greater clinical translation of research and collaboration between nations. Most importantly, there must be an increased effort to translate clinical trials in the Global South and create general accessibility to the researched medications and procedures. Being mindful of underdeveloped infrastructure, modifying research questions and adapting clinical methods to those that can be integrated easily with the existing health service may provide greater efficacy and value.

Although the implementation of any medical policy that has societal consequences is complicated by inefficiency, bureaucracy and corruption in many Global Southern countries, there are encouraging examples of bioethical development in South Africa, Nigeria, Kenya, Tanzania and the Democratic Republic of Congo.

## Conclusion

The issues that form the divide between the Global North and South are deeply rooted in historical sociopolitical factors and encompass neocolonial and postcolonial aspects of dependency and exploitation. These concepts suggest that medical globalization of clinical trials may be placing greater importance in scientific advancement than the welfare of the individuals involved, particularly in the developing world setting. Nevertheless, we have highlighted key areas that can be addressed to encourage future collaboration and improve the bioethical standards in the Global South. Solutions to the issues of medical globalization require a wide international input, ranging from global health institutions to the pharmaceutical industries. Most importantly, the Global South nations nations must be encouraged to take ownership of their bioethical agendas. We remain optimistic that with a global and persistent approach to this problem, the issue of medical neocolonialism and exploitation can be resolved.
